# Bayesian analysis of herd-level risk factors for bovine digital dermatitis in New Zealand dairy herds

**DOI:** 10.1186/s12917-019-1871-3

**Published:** 2019-04-27

**Authors:** Dan Aaron Yang, M. Carolyn Gates, Kristina R. Müller, Richard A. Laven

**Affiliations:** School of Veterinary Science, Massey University, Palmerston North, 4474 New Zealand

**Keywords:** Digital dermatitis, Dairy cattle, Lameness, Risk factors, Pastoral system, Bayesian, Multilevel modelling

## Abstract

**Background:**

Bovine digital dermatitis (BDD) is considered the most important infectious cause of lameness in dairy cattle worldwide, but has only recently been observed in New Zealand. Although many studies have investigated the risk factors for BDD in confined dairy systems, information on risk factors in pasture-based system is limited. Therefore a cross-sectional study including 59,849 animals from 127 dairy herds in four regions of New Zealand was conducted to identify the herd-level factors associated with the probability of a herd being BDD-lesion positive and with within-herd BDD prevalence.

**Results:**

Purchasing heifers was associated with increased odds of a herd being BDD-lesion positive (odds ratio [OR]: 2.33, 95% probability interval [PI]: 1.26–4.42) and a cow being BDD affected (OR: 3.76, 95%PI: 1.73–8.38), respectively. Higher odds of a herd being BDD-lesion positive (OR: 2.06, 95%PI: 1.17–3.62) and a cow being BDD affected (OR: 2.87, 95%PI: 1.43–5.94) were also seen in herds where heifers co-grazed with cattle from other properties. In addition, using outside staff to treat lameness was associated with higher odds of a cow being BDD affected (OR: 2.18, 95%PI: 0.96–4.98).

**Conclusion:**

This study highlighted that movements of heifers are significantly associated with the spread of BDD within and between dairy herds in New Zealand. To minimise the risk of disease introductions in herds where moving heifers cannot be avoided, it is best to purchase heifers only from herds where BDD-freedom has been confirmed and, if heifers have to graze-off a farm, they should be reared as a single biosecure management group, especially since animals may be BDD-infected without having clinically obvious lesions.

**Electronic supplementary material:**

The online version of this article (10.1186/s12917-019-1871-3) contains supplementary material, which is available to authorized users.

## Background

Bovine digital dermatitis (BDD) has been found throughout the world in both confined and pasture-based dairy systems [1, 2]. In many countries, BDD appears to be endemic in dairy herds [3] and is commonly considered as the most important infectious cause of cattle lameness [4]. Clinically, BDD lesions progress or regress through different morphological stages, commonly described using M scores [5, 6]. A rapid BDD lesion detection method such as visual examination during milking is widely used in many studies [7]; although interpretation of such diagnostic outcome is subjective, which usually requires additional validation studies to assess the agreement across the examiners [8].

Multiple studies have evaluated the risk factors associated with BDD prevalence within herds in confined dairy systems. These studies have identified a wide-range of potential risk factors including type of housing [9], using outside staff to trim hooves [10], footbath regimen [11] and access to pasture [12]. In contrast, very few studies [13–15] have been undertaken in cattle that are principally pasture-based with no or very limited use of housing, where many of the risk factors identified in confined animals are irrelevant. Specific research in such systems is essential as there can be large variation between pasture-based dairy herds in the prevalence of BDD [16].

In New Zealand, one previous study has evaluated herd-level risk factors for BDD, but that was undertaken in only one region [14]. In that study we used a Bayesian hurdle model to explore the associations between risk factors and BDD prevalence at both the herd and animal levels. The initial separation of the herds into BDD-lesion-free and BDD-lesion positive was based on whether BDD lesions were observed; i.e. a herd with ≥1 lesion was defined as being BDD-lesion positive, otherwise it was defined as being BDD-lesion free [14]. However, simply basing herd status on the presence/absence of visible lesions probably leads to loss of information regarding probability of a herd having BDD and may introduce misclassification bias at the herd level, as there is a chance that a herd where BDD lesions are truly present could be wrongly classified as being BDD-lesion-free due to a combination of limited diagnostic sensitivity and low cow-level prevalence [17].

One method for overcoming this limitation is by using a Bayesian latent class model, which estimates the mean probability of a herd being BDD-lesion positive conditional on the number of test positive animals, the total number of animals tested, and the test characteristics [17]. Thus, the mean probability contains more precise information than the simple dichotomised outcome and increases the power of the study to determine the impact of risk factors on the likelihood of a herd being BDD-lesion positive.

The aim of this study was to use Bayesian methods to investigate the impact of farm management practices on pasture-based dairy herds across New Zealand on 1) the probability of a herd being BDD-lesion positive obtained from a previous Bayesian latent class analysis [16] and 2) the within-herd BDD prevalence, namely the probability of a cow within a herd having BDD lesions.

## Methods

### Target and source population

The target population was the pasture-based dairy herds in New Zealand and the source population was the herds in the four regions across New Zealand: Waikato and Manawatu in the North Island and the West Coast and Canterbury in the South Island. These regions encompass most of the dairy systems (all grass fed and self-contained; feed imported, either supplement or grazing-off and feed imported to extend lactation) used in New Zealand [16].

### Data collection

The dataset was collected as described by Yang et al. [16]. Briefly, the data collection started in the Waikato and moved south following the seasonal pattern of calving to ensure that the great majority of the herds were milking at the herd examinations. In the first phase, half the sampled herds were visited in each region before moving on to the next. In the second phase, the order was reversed, starting in Canterbury and going back north. Within each herd, visual assessment was performed on cows’ rear feet in the milking parlour after hosing the feet gently [7].

The farm management practices undertaken in the previous 12 months were collected alongside the visual inspection for BDD using a questionnaire given to the owners or managers of the study herds. The questionnaire was modified by the authors from that used in Yang et al. [14]. The questionnaires were answered after the herd inspection while the first author was still on the farm, so that if the owners or managers were unsure of a question, the first author could explain the intent of the question. The categorical management predictors collected via the questionnaire are shown in Table 1 and a copy of the questionnaire is provided as an additional file (see Additional file 1).

**Table 1:** Herd-level predictors on BDD collected from 127 New Zealand dairy herds

Variable	Levels	Herds with BDD lesions *N* (%)	Herds without BDD lesions *N* (%)	Total
Type of milking parlour	Rotary	29 (51%)	28 (49%)	57
	Herringbone	34 (49%)	36 (51%)	70
Calving season	Spring only	57 (49%)	59 (51%)	116
	Spring and Autumn	6 (55%)	5 (45%)	11
Whether or not having dairy cattle milking on more than one farm	Yes	9 (14%)	6 (9%)	15
	No	54 (48%)	58 (52%)	112
Source of acquired adult cows (> 2 years old)	Other farms	8 (62%)	5 (38%)	13
	Saleyard	2 (50%)	2 (50%)	4
	Not acquiring	53 (48%)	57 (52%)	110
Source of acquired bulls	Other farms	36 (47%)	41 (53%)	77
	Saleyard	9 (64%)	5 (36%)	14
	Not acquiring	18 (50%)	18 (50%)	36
Source of acquired heifers	Other farms	15 (68%)	7 (32%)	22
	Saleyard	1 (50%)	1 (50%)	2
	Not acquiring	47 (46%)	56 (54%)	103
Whether your calves/heifers co-grazing with calves/heifers from other farms	Yes	46 (59%)	32 (41%)	78
	No	17 (35%)	32 (65%)	49
Whether your milking dairy cattle co-grazing with cows from other farms in winter	Yes	28 (57%)	21 (43%)	49
	No	35 (45%)	43 (55%)	78
Providing grazing for stock from other farms at your farm or not	Yes	3 (50%)	3 (50%)	6
	No	60 (50%)	61 (50%)	121
Using a transport company to transport animals (not for slaughter) or not	Yes	43 (49%)	45 (51%)	88
	No	20 (51%)	19 (49%)	39
Share a loading ramp or not	Yes	7 (41%)	10 (59%)	17
	No	56 (51%)	54 (49%)	110
Who did most of the hoof trimming/ lame cattle treatment on your farm	Vet	7 (39%)	11 (61%)	18
	Hoof trimmer	6 (86%)	1 (14%)	7
	On-farm staff	50 (49%)	52 (51%)	102
Trimming equipment cleaning methods	Washed by water	28 (51%)	27 (49%)	55
	Chemically disinfected	11 (55%)	9 (45%)	20
	Not wash	24 (46%)	28 (54%)	52
Use a footbath or not	Yes	10 (63%)	6 (37%)	16
	No	53 (48%)	58 (52%)	111
Whether or not use stand-off pads or cow houses in winter or poor weather	Stand-off pad	18 (49%)	19 (51%)	37
	Cow house	2 (22%)	7 (78%)	9
	Neither	43 (53%)	38 (47%)	81
Main material of the walking track/race from paddock to the milking parlour	Gravel	22 (35%)	40 (65%)	62
	Concrete	1 (50%)	1 (50%)	2
	Other	40 (63%)	23 (37%)	63
Use feedpad or not	Yes	23 (48%)	25 (52%)	48
	No	40 (51%)	39 (49%)	79

BDD, bovine digital dermatitis; *N* (%), numbers of herds having such a predictor (row percentage of herds having such a predictor)

Average herd milk solid production in BDD-lesion positive/negative herds were 414.9 kg/cow year and 414.1 kg/cow year, respectively

### Data processing

The data were imported into Stata 13.1 for cleaning and analysis (StataCorp, USA). One-way tables were used to examine the frequency of responses for each level within the categorical variables. Levels with low frequencies were combined with adjacent levels where biologically plausible. As hoof trimmers were rarely used to trim cows or treat lame cows, this level was combined with using vets to treat lame cows, to create a new dichotomous variable of whether or not the farm had outside staff trimming cows or treating lame cows. Since few farmers reported chemically disinfecting hoof trimming equipment, “chemical disinfection” and “washed by water” were combined to create a new variable of whether or not trimming equipment was cleaned between animals. Since few farmers reported purchasing dairy heifers or cows from saleyards, “saleyards” was combined with “other farms”, to create new variables of whether heifers and cows were purchased from outside. Similarly, cow houses were rarely used, so “cow house” was combined with “stand-off pad” and a new variable was created to describe whether the cows were permanently pasture-based (except for milking) or not. All categorical variables included in the final analysis had at least two levels and each level had at least ≥15% of the total responses for the question.

### Evaluating seasonal variation

As this was a cross-sectional study, the impact of season on BDD-lesion status and lesion prevalence was not of primary interest. However, as all the data were collected by the first author, it was not possible to complete the data collection in a short time frame and therefore herds were sampled at different points throughout the 2016/2017 lactation season. To confirm that BDD-lesion status and lesion prevalence did not vary significantly between different months, generalized estimating equations [18] and beta regression models were respectively used to examine whether the average cow-level prevalence or probabilities of a herd being BDD-lesion positive differed significantly between months in Waikato and Manawatu regions. This process was not applied to Canterbury since 18/19 herds were visited in the same month.

### Univariable models

For the two outcome variables (within-herd prevalence and probability of a herd being BDD-lesion positive), univariable logistic regression models and univariable beta regression models in the frequentist framework were respectively used to select predictors for fitting in the multivariable models. Any predictors with *p*-value ≤0.2 were included in the further analyses.

### Multivariable model 1

This analysis was designed to quantify the strength of associations between farm management practices and within-herd prevalence. A Bayesian binomial model was constructed. The model was built using a forward stepwise strategy. The predictors were retained in the model when the 90% probability intervals of their corresponding regression coefficients did not overlap 0. If inclusion of a predictor altered the coefficient of any of the existing predictors by > 15%; the newly included predictor was considered as a confounder and was forced into the model regardless its 90% probability interval [19]. Once the preliminary main effect model was constructed, two-way interactions between all predictors in the model were created. An interaction term was retained if its 95% probability interval excluded 0. The final model structure is presented below:

1 $$ {\displaystyle \begin{array}{l}{y}_j\kern0.5em \sim \kern0.5em binomial\left({p}_j,\kern0.5em {n}_j\right)\\ {}\mathrm{logit}\left({p}_j\right)\kern0.5em =\kern0.5em {\beta}_0\kern0.5em +\kern0.5em {\beta}_1{x}_j\kern0.5em +\kern0.5em {\beta}_2{\mathit{\mathsf{g}}}_j\kern0.5em +\kern0.5em {\beta}_3{h}_j\kern0.5em +\kern0.5em {U}_{region(j)}\kern0.5em +\kern0.5em {W}_j\\ {}{U}_{region(j)}\kern0.5em \sim \kern0.5em N\left(0,\kern0.5em {\sigma}_U\right)\\ {}{W}_j\kern0.5em \sim \kern0.5em N\left(0,\kern0.5em {\sigma}_W\right)\end{array}} $$

where *y*_*j*_ was the number of the cows with visible BDD lesions in the *j*^th^ herd of all the regions, which was modelled using a binomial distribution with the parameters: the proportion of cows with visible lesions (*p*_*j*_) and number of cows being examined (*n*_*j*_); *β*_0_ was the intercept, *β*_1_, *β*_2_ and *β*_3_ were the regression coefficients for the predictors *x*_*j*_, *g*_*j*_ and *h*_*j*_ which represented whether heifers were purchased from outside sources, whether heifers were co-grazed with heifers from other farms and whether outside personnel treated lame cows. Finally, *U*_*region*(*j*)_, *W*_*j*_ were the random effects at regional and herd level, respectively and modelled using two independent normal distributions with zero means and standard deviations *σ*_*U*_ and *σ*_*W*_.

The choice of prior distributions contributes to the posterior distributions, thus utilising informative priors results in better inferences compared to “vague” priors [20]. It is difficult to place informative priors for the regression coefficients. However, such priors can be indirectly induced to define probabilities for different combinations of predictors. Partially informative priors were assigned to *β*_0_, *β*_1_ and *β*_2_. First, the proportion of cows with visible lesions (prevalence) in a “typical” closed herd was defined as ${\overset{\sim }{p}}_0$. This meant *x* = 0, *g* = 0 and *h* = 0. Therefore, according to Eq. 1, ${\beta}_0=\mathrm{logit}\ \left({\overset{\sim }{p}}_0\right)$. Second, specify ${\overset{\sim }{p}}_1$ as the prevalence of a herd where some heifers were purchased from outside, in this case, *x* = 1, but *g* = 0 and *h* = 0. Thus, ${\beta}_1=\mathrm{logit}\ \left({\overset{\sim }{p}}_1\right)-{\beta}_0$. Finally, let ${\overset{\sim }{p}}_2$ denote the prevalence of a herd which contained purchased heifers, and, at the same time, sent its own heifers to co-graze with heifers from other farms (*x* = 1 and *g* = 1, but *h* = 0). This gave ${\beta}_2=\mathrm{logit}\ \left({\overset{\sim }{p}}_2\right)-\mathrm{logit}\ \left({\overset{\sim }{p}}_1\right)$. Logit-normal distribution was used for these prevalence priors. Below we use ${\overset{\sim }{p}}_0$ as an example to illustrate the way to convert a prevalence estimate to its corresponding logit-normal distribution such as $\mathrm{logit}\ \left({\overset{\sim }{p}}_0\right)\sim N\left({\mu}_{\beta 0},{\tau}_{\beta 0}\right)$, where *τ* is the precision term defined as the reciprocal of the variance. Our best estimate of the prevalence in a closed herd was *m*_0_ and we were 95% confident that it was less than *l*_0_; then *μ*_*β*0_ = logit(*m*_0_). The standard deviation *σ*_*β*0_ = [logit(*l*_0_) − logit(*m*_0_)]/1.645, eventually ${\tau}_{\beta 0}=1/{\sigma}_{\beta 0}^2$.

The best estimates of ${\overset{\sim }{p}}_0$, ${\overset{\sim }{p}}_1$ and ${\overset{\sim }{p}}_2$ came from the previous analyses of BDD data in Taranaki and the authors’ expert opinion. One important observation was that in contrast to previous studies of housed cattle, the apparent cow-level prevalence of BDD was very low (mean = 1.2%, Yang et al. [1]) with 26.8% of herds having fewer than1% of cows with observed lesions. In Canterbury region, where median herd size was 840 and > 21% of herds had ≥1000 cows; we were able to detect BDD at an apparent within-herd prevalence of 0.1% (i.e. one cow with lesions in a 1000-cow herd). Thus to reflect our belief that a closed herd was likely to have no or extremely rare BDD lesions, we took 0.05% as our “best point estimate” for ${\overset{\sim }{p}}_0$. Furthermore we were also 95% confident that it was less than 0.35%, i.e., one cow with BDD lesion(s) in a 300-cow herd. Based on the method described in the last paragraph, this led to *μ*_*β*0_ =  − 7.6 and *τ*_*β*0_ = 0.71. Table 2 summarises our “best estimates” for ${\overset{\sim }{p}}_0$, ${\overset{\sim }{p}}_1$ and ${\overset{\sim }{p}}_2$. Uniform priors (0, 3) and (0, 2) were set for *σ*_*U*_ and *σ*_*W*_, respectively. This reflected our belief that the variability of herd-level prevalence across regions was bigger than the variability across herds. However, the parameter values assigned to the uniform priors were considered to be non-specific as we did not know the standard deviations of the two random effects.

**Table 2:** The “best estimates” for within-herd prevalence (${\overset{\sim }{p}}_k$) of BDD conditional on the different covariates

			Prevalence	
	Purchasing heifers	Heifers co-grazing	prior mode	95th percentile
${\overset{\sim }{p}}_0$	No	No	0.05%	0.35%
${\overset{\sim }{p}}_1$	Yes	No	0.2%	1%
${\overset{\sim }{p}}_2$	Yes	Yes	0.5%	3%

BDD, bovine digital dermatitis

Under the partially informative priors, the fit of the model to the data was evaluated using posterior predictive checks which compared the observed outcome data to the data simulated/predicted by the posterior predictive distribution [21]. The Bayesian *P*-value quantifies the probability that the discrepancy between the predicted and observed values. A Bayesian P-value close to 0.50 indicates adequate model fit, although a value between 0.20 and 0.80 is also accepted [22].

Sensitivity analysis was used to assess the sensitivity of the posteriors to the priors. Table 3 summarises the distributions of the model priors and the priors used for the sensitivity analysis. The model was developed using OpenBUGS [23]. Posterior inferences were obtained using Markov chain Monte Carlo (MCMC) approximation. The posterior distribution of each parameter was reported using median and 95% probability interval (PI). After discarding the first 10,000 iterations as burn-in period, the model was further run for 100,000 iterations. Convergence was assessed using BGR-plots by running three chains with different sets of initial values [24].

**Table 3:** The prior distributions for parameters used in the Bayesian multilevel multivariable binomial model

Parameter	Main analysis	Sensitivity analysis scenarios		
		1	2	3
*β*_0_	*N* (−7.6, 0.71)	*N* (−5, 0.001)	*N* (−7.6, 0.71)	*N* (−7.6, 0.71)
*β*_1_	$\mathrm{logit}\left({\overset{\sim }{p}}_1\right)-{\beta}_0$	*N* (0, 0.001)	$\mathrm{logit}\left({\overset{\sim }{p}}_1\right)-{\beta}_0$	$\mathrm{logit}\left({\overset{\sim }{p}}_1\right)-{\beta}_0$
*β*_2_	$\mathrm{logit}\left({\overset{\sim }{p}}_2\right)-\mathrm{logit}\left({\overset{\sim }{p}}_1\right)$	*N* (0, 0.001)	$\mathrm{logit}\left({\overset{\sim }{p}}_2\right)-\mathrm{logit}\left({\overset{\sim }{p}}_1\right)$	$\mathrm{logit}\left({\overset{\sim }{p}}_2\right)-\mathrm{logit}\left({\overset{\sim }{p}}_1\right)$
*β*_3_	*N* (0, 0.001)	*N* (0, 0.001)	*N* (0, 0.001)	*N* (0, 0.001)
*σ*_*U*_	Uniform (0, 3)	Uniform (0, 3)	Uniform (0, 5)	Uniform (0, 9)
*σ*_*W*_	Uniform (0, 2)	Uniform (0, 2)	Uniform (0, 3)	Uniform (0, 2)

*β*_0_, intercept; *β*_1_, purchasing heifers; *β*_2_, heifers co-grazing; *β*_3_, lameness treated by outside staff

*σ*_*U*_ and *σ*_*W*_, standard deviation of random effects at region and herd levels

${\overset{\sim }{p}}_1$ ~ logit-normal (−6.21, 1.03); ${\overset{\sim }{p}}_2$ ~ logit-normal (−5.29, 0.82)

### Multivariable model 2

This analysis was designed to assess the associations between farm management predictors and the probability of herd being BDD-lesion positive (PP). This analysis did not include herds on the West Coast as the region was determined to be free of the disease [16].

The data were modelled using a Bayesian beta model [25]. *π*_*k*_ was used to denote the PP_*k*_ for *k*^th^ herd . The variable “region” was initially modelled as a random effect ${V}_{region(k)}\sim N\left(0,{\frac{1}{\sqrt{\tau}}}_V\right)$, where *τ*_*V*_ was the precision term. The model was constructed as follows:

$$ {\pi}_k\sim Beta\left({a}_k,{b}_k\right) $$

2 $$ {a}_k={\mu}_k\varphi $$

3 $$ {b}_k=\varphi \left(1-{\mu}_k\right) $$

4 $$ \mathrm{logit}\left({\mu}_k\right)=\gamma {z}_k+{V}_{region\;(k)} $$

$$ {V}_{region(k)}\sim N\left(0,\frac{1}{\sqrt{\tau_V}}\right) $$

where *z*_*k*_ was the predictor vector, *γ* denoted the regression coefficient vector and *μ*_*k*_ the mean and *φ* a measure of variability, with a larger value of *φ* indicating less variability [26].

Diffuse normal distributions (mean = 0, precision = 0.01) were set for all the regression coefficients, and a vague gamma distribution (1, 1) was set for *φ* and *τ*_*V*_. The model was built using a forward stepwise strategy. Predictors were retained if the 90% probability interval for the regression coefficients excluded 0. Confounders were assessed using the method described as per Multivariable model 1. Two-way interactions between all predictors in model were investigated after building the main effect model. Inclusion criteria for an interaction term were the same as for Multivariable model 1. In this model, the linear predictor was the log-odds. The odds were defined as the probability of a herd being BDD-lesion positive divided by the probability of a herd being BDD-lesion-negative at each level of a predictor. The model was therefore able to identify any farm management practice associated with higher odds of being BDD-lesion positive for a randomly selected herd in any BDD-affected region.

### Multivariable model 3

Although the mixed effects beta model modelled the overall variability of the probability in different regions; it was not able to describe the difference between particular regions, therefore we also built a model which treated “region” as a fixed effect. Assuming the model had in total *t* farm management practices, Eq. (4) was changed to:

5 $$ \mathrm{logit}\left({\mu}_k\right)={\gamma}_c{z}_{ck}+{\gamma}_w{z}_{wk}+{\gamma}_1{z}_{1k}+\dots +{\gamma}_t{z}_{tk} $$

with ${V}_{region(k)}\sim N\left(0,{\frac{1}{\sqrt{\tau}}}_V\right)\ \mathrm{dropped}$. Here, *z*_*ck*_ and *z*_*wk*_ were the dummy variables for the regions Canterbury and Waikato (level “Manawatu” was treated as reference level). This fixed effects model can be used to predict the probability of a herd being BDD-lesion positive with different covariates in any particular region.

The deviance information criteria (DIC) of both beta models were compared. In addition, a global measure of variation explained by each of the beta models was obtained by computing pseudo-R^2^ defined as the squared correlation between the linear predictor and the logit-transformed outcome variable [27]. Both beta models were developed using OpenBUGS [23]. After discarding the first 5000 iterations as the burn-in period, the model was further run for 100,000 iterations. Convergence was assessed using BGR-plots by running three chains with different sets of initial values [24]. The OpenBUGS code for Multivariable model 1, 2 and 3 is provided as an additional file (see Additional file 2).

## Results

There was no evidence to support seasonal differences in any of the outcome variables. In Waikato region, the average cow-level prevalences in September 2016 and in January 2017 were not significantly different (*P* = 0.94). The probabilities of BDD-lesion positive also did not differ significantly between these two months (*P* = 0.65). In Manawatu, the average cow-level prevalences in September (*P* = 0.46) and November (*P* = 0.22) were not significantly different to that in December. Similarly, significant differences in the probabilities of BDD-lesion positive in September (*P* = 0.86), November (*P* = 0.28) and December were not evident. These findings ruled out the potential seasonal impact on BDD prevalences in this study. Table 4 displays the total herds and animals sampled as well as the proportions of herds/animals having BDD lesions in each region during the data collecting period.

**Table 4:** Total (#) herds/cattle sampled, proportions (%) of herds/cattle with BDD lesions detected in each region

	Region			
Parameters	Waikato	Manawatu	The West Coast	South Canterbury
# of herds	40	41	27	19
# and % of affected herds	34 (85%)	15 (37%)	0	14 (74%)
# of cows	15,522	15,546	12,978	15,803
# and % affected cows	241 (1.6%)	68 (0.4%)	0	337 (2.1%)

*BDD* bovine digital dermatitis

The outputs from the Bayesian binomial model (Multivariable model 1) with our partially informative priors are shown in Table 5. Lack of model fit was not evident (Bayesian *P*-value = 0.5). The posteriors for *β*_1_, *β*_2_ and *β*_3_ were robust in all sensitivity analysis scenarios. The posterior median for *β*_0_ increased slightly (− 8.05 vs. − 7.67) and its 95%PI was also wider (− 10.65, − 5.707) given the diffuse prior *N* (− 5, 0.001) rather than the informative prior. The posterior for *σ*_*W*_ was not sensitive to its prior, although the posterior for *σ*_*U*_ was sensitive to its prior. The posterior median of *σ*_*U*_ increased from 2.4 to 3.2 when the prior changed from Uniform (0, 3) to Uniform (0, 5). It further increased to 4 if the prior changed to Uniform (0, 9). Nevertheless, there was no impact on the posteriors for the regression coefficients. The results of the sensitivity analyses are provided as an additional file (see Additional file 3).

**Table 5:** The posterior distributions for parameters of the Bayesian multilevel multivariable binomial model

Parameter	Interpretation	Posterior distribution		
		Median	2.5th Percentile	97.5th Percentile
*β*_0_	Intercept	-7.67	−8.9	−6.46
*β*_1_	Purchasing heifers	1.32	0.55	2.13
*β*_2_	Heifers co-grazing	1.06	0.36	1.78
*β*_3_	Lameness treated by outside staff	0.78	−0.04	1.61
*σ*_*U*_	Region level random effect	2.36	1.33	2.97
*σ*_*W*_	Herd level random effect	1.41	1.12	1.8

Based on Multivariable model 1, cattle in a herd which purchased heifers from outside were more likely to have BDD lesions than cattle in a herd that did not purchase heifers (OR: 3.76, 95%PI: 1.73–8.38). Being in a herd which co-grazed heifers with animals from other properties also increased the odds of a cow having BDD lesions (OR: 2.87, 95%PI: 1.43–5.94). The use of outside staff to treat lameness was found to be associated with the increased within-herd prevalence (OR: 2.18, 95%PI: 0.96–4.98).

**Fig. 1:**
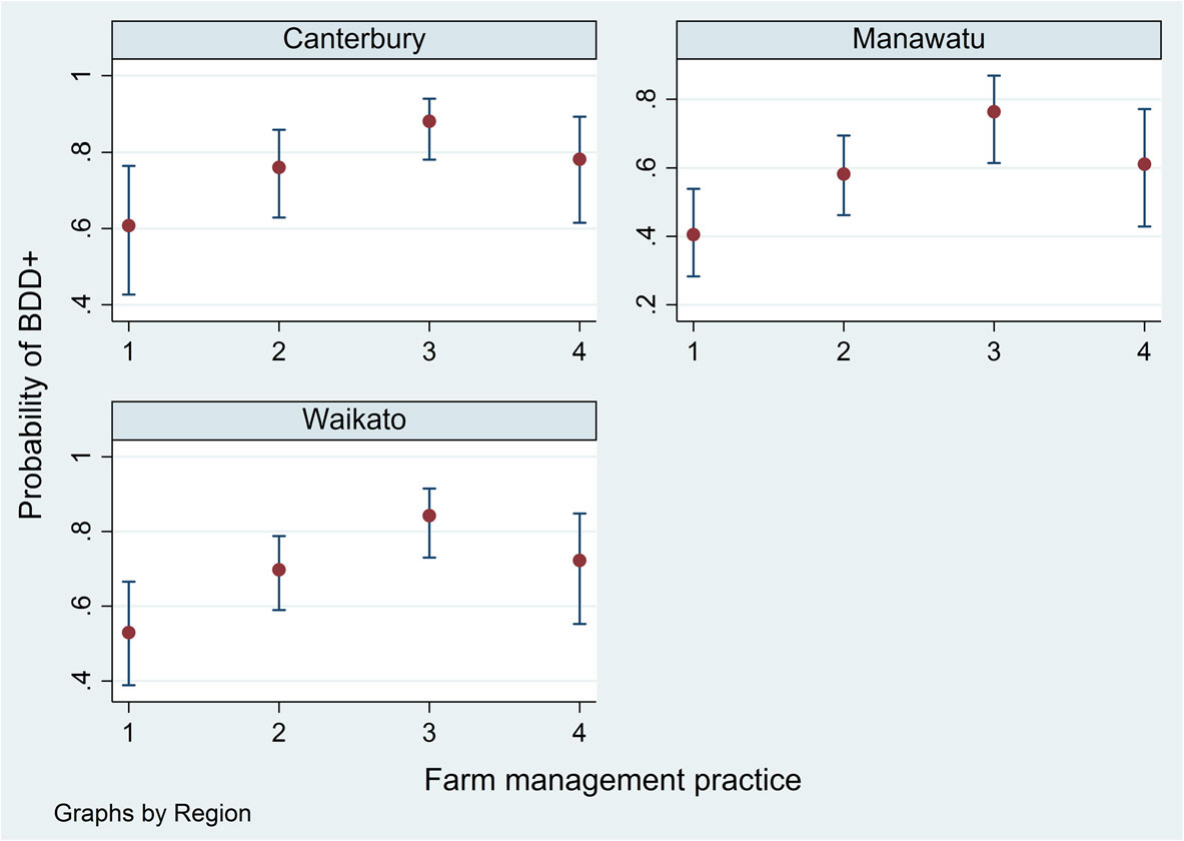
Predicted probability of a herd being BDD-lesion positive given different farm management practices. 1 = a closed herd, 2 = a herd having heifers co-grazing with animals from other properties only, 3 = a herd having heifers co-grazing with animals from other properties and having purchased heifers, 4 = a herd having purchased heifers only; BDD = bovine digital dermatitis

Except for the intercepts, the posteriors for the parameters reported by the Bayesian mixed effects beta model and fixed effects beta model were nearly identical. Table 6 summarises the models’ outputs. The DIC for each model was also very similar, − 533.3 for the fixed effects model and − 533.5 for the mixed effects model. Two farm management practices were identified as being significantly associated with the odds of a herd being BDD-lesion positive. Based on the mixed effects beta model, the odds of a herd being BDD-lesion positive was 2.33 times (95%PI: 1.26–4.42) higher in a herd with purchased heifers compared to one without, and 2.06 times (95%PI: 1.17–3.62) higher if heifers co-grazed with cattle from other properties. The predicted probabilities from the fixed effects model of a herd being BDD-lesion positive conditional on different farm management practices and region are displayed in Fig. 1. However, 74% of the variation in the probability of a herd being BDD-lesion positive remained unexplained by either beta model (pseudo-R^2^ = 0.26).

**Table 6:** The posterior distributions for parameters of the Bayesian beta models

Parameter		Posterior distribution		
		Median	2.5th Percentile	97.5th Percentile
*Mixed effects beta model*				
*γ*_0_	Intercept	0.07	−1.13	1.36
*γ*_1_	Heifers co-grazing	0.73	0.16	1.29
*γ*_2_	Purchasing heifers	0.85	0.23	1.49
*φ*		0.53	0.42	0.66
*τ*_*V*_		1.43	0.22	4.81
*Fixed effects beta model*				
*γ*_0_	Intercept	−0.38	−0.93	0.16
*γ*_1_	Heifers co-grazing	0.72	0.15	1.28
*γ*_2_	Purchasing heifers	0.84	0.22	1.48
*γ*_*c*_	Canterbury	0.82	0.11	1.55
*γ*_*w*_	Waikato	0.50	−0.08	1.09
*φ*		0.53	0.42	0.66

*φ* = *a* + *b*, where *a* and *b* are shape parameters of a beta distribution

*τ*_*V*_, precision term of the region level random effect defined as 1/variance

## Discussion

This study found that both co-grazing with heifers from other properties and purchasing heifers from other farms were associated with an increased probability of a herd being BDD-lesion positive as well as increased within-herd prevalence. Our previous study [14] also found that that youngstock movement between farms considerably increased the probability of a farm having at least one visible lesion (OR: 4.15, 95%PI: 1.39–15.27). Compared to Yang et al. [14], our current study evaluated youngstock movement in a more detailed way by dividing such movements into heifer purchasing and heifer co-grazing. Unlike Yang et al. [14] who reported that youngstock movement affected only a herd’s probability of having at least one cow with BDD lesions but not within-herd prevalence, this broader-scale study (along with the more detailed way of recording the predictors) found that youngstock movement increased both the probability of a herd being BDD-lesion positive and the within-herd prevalence. The likely inference is that heifers act as a reservoir for BDD transmission between dairy herds and between cows within herds in New Zealand [28]. In contrast, both this analysis and Yang et al. [14] found no effect of purchasing adult cattle on BDD risk. This lack of effect is most likely due to the much smaller numbers of purchased adult cows compared to the numbers of heifers purchased for replacement [13, 14].

Yang et al. [14] reported that herds with a rotary platform were more likely to have at least one cow with BDD lesions than herds with a herringbone (OR: 3.19, 95%PI: 1.31–8.51), though as with heifer movement, no effect was seen on within-herd prevalence. This may have been due to the ease of finding at least one lesion in herds with rotary platforms rather than being an actual risk factor [14]. Our current analysis did not include parlour type in the final model as the analysis found it to be neither statistically significant nor a confounder.

Two New Zealand studies [14, 15] reported that on BDD-positive farms, the within-herd prevalence was higher on farms where the outside staff came for hoof trimming (prevalence ratio [PR]: 3.13, 95%PI: 1.25–7.29 and risk ratio [RR]: 2.06, 95% confidence interval [CI]: 1.05–4.06). Although our current analysis did not confirm this finding, the calculated OR 2.18 was still in the realm considered to be biologically important [10] and the 95%PI: 0.96–4.98 only just included 1. It is not entirely clear how these effects could be mediated under New Zealand conditions. The use of outside staff for lame cows is typically unrelated to BDD since BDD rarely causes lameness in New Zealand dairy cattle. However, failure to clean trimming equipment properly between cows and between herds could represent a mechanism for spread [14]. To confirm this hypothesis, our current study included whether trimming equipment was cleaned between cows as a potential risk factor. However, no effect of cleaning/washing equipment between cows was found; this suggests that if there is an effect of outside staff on the within-herd prevalence of BDD that it is not mediated via dirty equipment. Further research is required to better estimate the impact of using outside staff to treat lame cows on BDD prevalence and to investigate potential pathways by which such an effect could be mediated.

The only other study of risk factors for BDD in pasture-based cows is that by Rodriguez-Lainz et al. [13]. However, of the 22 farms in that study only 2 kept their cattle at pasture all year round, with 13/22 keeping cattle in an open corral or loose yard for at least part of the year, whereas in this study, all 127 farms grazed their cattle throughout the year. As such many of the factors analysed by Rodriguez-Lainz et al. [13] (e.g. housing type and season of calving) are not directly relevant to the New Zealand situation and thus not included in our analysis. Although it is difficult to directly compare the study findings, Rodriguez-Lainz et al. [13] did find that there was an effect of purchasing replacement heifers on within-herd prevalence of BDD (OR: 3.16, 95%CI: 1.61–6.21), but not purchasing adult cows (OR: 1.31, 95% CI: 0.72–2.38). The data from Rodriguez-Lainz et al. [13] provided no evidence as to whether, in pasture-based cattle, using outside staff to trim feet increases the within-herd prevalence of BDD as in that study all cattle were treated or trimmed by farm staff. However, using hoof trimmers who operated on multiple farms was found to be significantly associated with higher BDD within-herd incidence in housed cattle (OR: 2.8, 95% CI: 1.9–4.2) [10].

Many studies on dairy cattle from intensive housing systems in the northern hemisphere have also identified herd-level risk factors for this disease. Decreasing the access to pasture was found to increase the risk of BDD [12, 29, 30]. The type of housing for animals was also associated with BDD prevalence, i.e. cows that housed in cubicles had higher BDD prevalence and more severe BDD cases [31] than cows in straw yards, which also agreed with Onyiro et al. [9]. In cubicles houses, the size of cubicles was linked to the risk of BDD [29]. This is because cows tend to spend longer time standing in shorter and narrower cubicles; therefore the contact between heels and slurry was increased [32]. However, these factors tend not to be an issue in New Zealand pasture-based systems and were therefore not included in the current study. It could be interesting in future studies to evaluate cleanliness of legs in cattle since higher prevalence of BDD had been found in cows with dirty legs [33]. This is one possible explanation why no BDD lesions were seen on the West Coast where the cows’ feet were generally much cleaner compared to other regions.

The results of this study show that even in New Zealand where BDD prevalence is very low, heifers are the most likely source of disease spread between and within herds. Particular care should be taken when purchasing heifers as replacement animals and ideally, replacement heifers should only be purchased from herds where BDD-freedom has been confirmed. The latter may be difficult in New Zealand since many heifers are purchased in late-autumn when cows are not being milked and it is therefore not possible to observe the milking herd for BDD. In such cases, visual inspection of the whole heifer group (not just the heifers for purchase) is a potential alternative to increase the probability of finding at least one animal with BDD lesions. If any of the heifers have visible lesion(s), then the entire group should not be purchased as animals can still be infected with BDD in the absence of visible lesions [34]. Where heifers are co-grazing with animals from multiple herds, it becomes much more difficult to ensure that co-grazing heifers will not come in contact with BDD infected cattle, although little is currently known about the transmission dynamics of BDD in grazed dry stock. Thus, the only reliable method to ensure that heifers grazed away from the farm do not become infected with BDD is to require that they are grazed as a single biosecure management group. This is important to prevent the spread of many infectious diseases as well as BDD.

Bayesian methods were adopted as the analytical approach in this paper. Bayesian analyses incorporate previous scientific understanding, e.g. such as the likely association between a farm management practice and BDD within-herd prevalence, into analysis (see Multivariable model 1), so that the inference (i.e. the posterior distribution) is based on both the data and our prior information. This is in contrast to other methods which typically ignore such previous understanding [35]. Furthermore even if previous information of a research question is not available, the Bayesian methods still has significant advantages such as being able to directly compare the relative probabilities of two or more hypotheses rather than simply using the probability of the data given the null hypothesis to determine whether an alternative hypothesis was plausible.

Multivariable model 2 and 3 used uniform priors, as this was the first use of beta models to study risk factors on the herd-level BDD outcome estimated from a previous Bayesian latent class analysis. This use of the outcome from the latent class analysis reduced the likelihood of misclassification errors at the herd level, as the effect of diagnostic sensitivity and specificity on the herd level diagnosis was factored into the latent class model [16].

Although misclassification bias has been adjusted at the herd level in Multivariable model 2 and 3, our Multivariable model 1 did not account for animal level misclassifications. This could potentially have influenced the analysis of risk factors affecting within-herd prevalence. Misclassification at the individual level, as at the herd level, can be minimised by incorporating the known sensitivity and specificity of a diagnostic method [36]. However, when the impact of specificity and sensitivity on the diagnosis of BDD in the individual animal was included during the modelling process, it resulted in non-convergence of the Markov chains. This may be related to the model being non-identifiable. Using a more sensitive detection method inspecting lifted cows’ feet in the trimming chute, would have decreased any potential impact but would have been cost prohibitive [7].

The other limitation was that Multivariable models 2 and 3 explained only 26% of the variation in the probability of a herd being BDD-positive. This indicates that further investigation of more factors which could potentially affect the probability of herd being BDD-lesion positive was required.

## Conclusions

Our study investigated potential risk factors for BDD across New Zealand and identified that purchasing replacement heifers and co-grazing heifers with animals from other herds were significantly associated with a higher probability of a herd being BDD-lesion positive and higher within-herd prevalence of BDD. This is consistent with previous findings from pasture-based systems. However, the identified risk factors only explained a small proportion of the variation in probability of a herd being BDD-lesion positive. Our study also found that using outside staff for trimming had a large effect on within-herd prevalence (doubling the odds of an individual cow having BDD). Given that we can’t rule out the possibility of contaminated hoof trimming equipment contributing to the between-herd spread of BDD, it would be advisable for farms to maintain their own set of equipment. Further research should be undertaken to better estimate the impact of this factor on BDD and how it can be mediated through different biosecurity interventions.

## Additional files

Additional file 1: The questionnaire used to collect farm management practices. (DOCX 176 kb)

Additional file 2: OpenBUGS code for the three Bayesian multivariable models used in this study. (DOCX 19 kb)

Additional file 3: Results of the sensitivity analysis for Multivariable model 1. (DOCX 18 kb)

## Abbreviations

BDD: Bovine digital dermatitis
CI: Confidence interval
DIC: Deviance information criteria
MCMC: Markov chain Monte Carlo
OR: Odds ratio
PI: Probability interval
PP: Probability of herd being BDD-lesion positive
PR: Prevalence ratio
RR: Risk ratio

## References

[1] Yang DA, Heuer C, Laven R, Vink WD, Chesterton RN (2017). Farm and cow-level prevalence of bovine digital dermatitis on dairy farms in Taranaki, New Zealand. N Z Vet J.

[2] Solano L, Barkema HW, Mason S, Pajor EA, LeBlanc SJ, Orsel K (2016). Prevalence and distribution of foot lesions in dairy cattle in Alberta, Canada. J Dairy Sci.

[3] Orsel K, Plummer P, Shearer J, De Buck J, Carter S, Guatteo R, Barkema H (2018). Missing pieces of the puzzle to effectively control digital dermatitis. Transbound Emerg Dis.

[4] Laven R, Lawrence K (2006). An evaluation of the seasonality of veterinary treatments for lameness in UK dairy cattle. J Dairy Sci.

[5] Döpfer D, Koopmans A, Meijer F, Szakall I, Schukken Y, Klee W, Bosma R, Cornelisse J, Van Asten A, Ter Huurne A (1997). Histological and bacteriological evaluation of digital dermatitis in cattle, with special reference to spirochaetes and campylobacter faecalis. Vet Rec.

[6] Berry SL, Read DH, Famula TR, Mongini A, Döpfer D (2012). Long-term observations on the dynamics of bovine digital dermatitis lesions on a California dairy after topical treatment with lincomycin HCl. Vet J.

[7] Yang DA, Laven RA (2019). Detecting bovine digital dermatitis in the milking parlour: to wash or not to wash, a Bayesian superpopulation approach. Vet J.

[8] Yang DA, Laven RA (2019). Inter-observer agreement between two observers for bovine digital dermatitis identification in New Zealand using digital photographs. N Z Vet J.

[9] Onyiro O, Andrews L, Brotherstone S (2008). Genetic parameters for digital dermatitis and correlations with locomotion, production, fertility traits, and longevity in Holstein-Friesian dairy cows. J Dairy Sci.

[10] Wells S, Garber L, Wagner B (1999). Papillomatous digital dermatitis and associated risk factors in US dairy herds. Prev Vet Med.

[11] Speijers M, Baird L, Finney G, McBride J, Kilpatrick D, Logue D, O’Connell N (2010). Effectiveness of different footbath solutions in the treatment of digital dermatitis in dairy cows. J Dairy Sci.

[12] Holzhauer M, Brummelman B, Frankena K, Lam T (2012). A longitudinal study into the effect of grazing on claw disorders in female calves and young dairy cows. Vet J.

[13] Rodriguez-Lainz A, Melendez-Retamal P, Hird DW, Read DH, Walker RL (1999). Farm-and host-level risk factors for papillomatous digital dermatitis in Chilean dairy cattle. Prev Vet Med.

[14] Yang DA, Laven RA, Heuer C, Vink WD, Chesterton RN (2018). Farm level risk factors for bovine digital dermatitis in Taranaki, New Zealand: an analysis using a Bayesian hurdle model. Vet J.

[15] Yang DA, Laven RA, Chesterton RN (2019). Effects of climate and farm management practices on bovine digital dermatitis in spring-calving pasture-based dairy farms in Taranaki, New Zealand. Vet J.

[16] Yang DA, Johnson WO, Müller KR, Gates MC, Laven RA (2019). Estimating the herd and cow level prevalence of bovine digital dermatitis on New Zealand dairy farms: a Bayesian superpopulation approach. Prev Vet Med.

[17] Yang DA, Heuer C, Laven R, Vink WD, Chesterton RN (2017). Estimating the true prevalence of bovine digital dermatitis in Taranaki, New Zealand using a Bayesian latent class model. Prev Vet Med.

[18] Zeger SL, Liang K-Y, Albert PS (1988). Models for longitudinal data: a generalized estimating equation approach. Biometrics.

[19] Dohoo I, Martin W, Stryhn H: Veterinary Epidemiologic Research, 2nd edn. Charlottetown, Canada: VER Inc; 2010.

[20] Dunson DB (2001). Commentary: practical advantages of Bayesian analysis of epidemiologic data. Am J Epidemiol.

[21] Gelman A, Meng X-L, Stern H. Posterior predictive assessment of model fitness via realized discrepancies. Stat Sin. 1996:733–60.

[22] Neelon BH, O’Malley AJ, Normand S-LT: A Bayesian model for repeated measures zero-inflated count data with application to outpatient psychiatric service use. Stat Model 2010, 10(4):421–439. 10.1177/1471082X0901000404 PMC3039917 21339863

[23] Spiegelhalter D, Thomas A, Best N, Lunn D (2007). OpenBUGS user manual, version 3.0. 2.

[24] Brooks SP, Gelman A (1998). General methods for monitoring convergence of iterative simulations. J Comput Graph Stat.

[25] Branscum AJ, Johnson WO, Thurmond MC (2007). Bayesian beta regression: applications to household expenditure data and genetic distance between foot-and-mouth disease viruses. Aust N Z J Stat.

[26] Branscum A, Gardner I, Johnson W (2004). Bayesian modeling of animal-and herd-level prevalences. Prev Vet Med.

[27] Ferrari S, Cribari-Neto F (2004). Beta regression for modelling rates and proportions. J Appl Stat.

[28] Laven R, Logue D (2007). The effect of pre-calving environment on the development of digital dermatitis in first lactation heifers. Vet J.

[29] Somers J, Frankena K, Noordhuizen-Stassen E, Metz J (2005). Risk factors for digital dermatitis in dairy cows kept in cubicle houses in the Netherlands. Prev Vet Med.

[30] Read DH, Walker RL (1998). Papillomatous digital dermatitis (footwarts) in California dairy cattle: clinical and gross pathologic findings. J Vet Diagn Investig.

[31] Laven R (1999). The environment and digital dermatitis. Cattle Practice.

[32] Laven R: Determination of the factors affecting the cause, prevalence and severity of digital dermatitis as a major cause of lameness in dairy cows. Milk Dev Counc Study 2000, 95(May):1–5.

[33] Relun A, Lehebel A, Bruggink M, Bareille N, Guatteo R (2013). Estimation of the relative impact of treatment and herd management practices on prevention of digital dermatitis in French dairy herds. Prev Vet Med.

[34] Vink W, Jones G, Johnson W, Brown J, Demirkan I, Carter S, French N (2009). Diagnostic assessment without cut-offs: application of serology for the modelling of bovine digital dermatitis infection. Prev Vet Med.

[35] Johnson WO (2013). Comment: Bayesian statistics in the twenty first century. Am Stat.

[36] McGlothlin A, Stamey JD, Seaman JW (2008). Binary regression with misclassified response and covariate subject to measurement error: a Bayesian approach. Biom J.

