# Supplementary material for: Bayesian analysis of herd-level risk factors for bovine digital dermatitis in New Zealand dairy herds
Source: BMC Vet Res. 2019 Apr 27;15:125. doi: 10.1186/s12917-019-1871-3 (PMC6487038; doi:10.1186/s12917-019-1871-3)
Supplement: Supplementary file 1 — The questionnaire used to collect farm management practices. (DOCX 176 kb) [file 12917_2019_1871_MOESM1_ESM.docx]

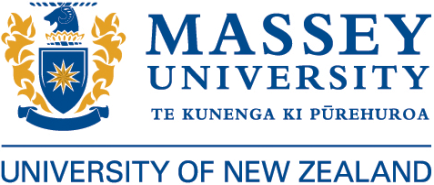

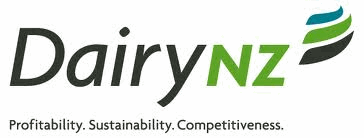


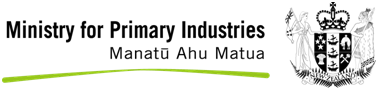


#### Bovine Digital Dermatitis in cattle:

#### Confidential farm management questionnaire

**2016-2017**

# ------------------------------------------------------------------------------------------------------------------

**This questionnaire should take 15-20 minutes to complete and your cooperation is much appreciated. This survey is being performed in conjunction with a herd screening for Bovine Digital Dermatitis (BDD). The aims are to describe management practices that may be relevant to BDD.**

Aaron Yang

IVABS, Massey University

Tennent Dr,

Palmerston North 4474

For any queries, please contact [d.yang@massey.ac.nz](mailto:d.yang@massey.ac.nz)

**BDD in New Zealand**

BDD has not been observed in New Zealand until 2004. From 2004 to 2011, sporadic cases were reported to Ministry for Primary Industries. In 2014, a large scale cross-sectional survey in Taranaki raised awareness of the disease. There were no outbreaks in any herd but more than 60% of herds were infected. The concern is that the disease may start spreading as it has done elsewhere in the world and contribute to incidence of lameness on farm. We therefore urgently require more information of this disease across the country to investigate the prevalence and to identify farm management practices which could at some point trigger epidemic outbreaks of BDD.

To date, we do not have a strong scientific understanding of the dynamics of the disease. Our objectives are to

- provide scientifically-based evidence to assess whether the disease is spreading or becoming more common in New Zealand;
- characterise the cases identified in New Zealand, and describe the patterns of disease within herds;
- develop relevant protocols and recommendations for identifying, treating and controlling the disease.

### Confidentiality

All information given will be treated with confidentiality. The farm details recorded on this page will be accessible to the principal researchers only. We will not contact you without your consent, and even if given, will not contact you unless necessary.

# Farm details

1. Please complete the following contact details:

| Name and farm address | _________________________________________________________________________________________________________________________________ |
| --- | --- |
| Email address | __________________________ |
| Farm telephone / mobile | Tel. _______________________  Mobile_____________________ |
| FarmsOnLine number (if applicable) | __________________________ |
| Person completing the questionnaire | - farm owner(s) - farm manager(s) - stockperson(s) - other: ______________________________________ |
| Date of completion | ____ / ____ / ______ |
| Veterinary practice | ___________________________________________ |

2. Which region are you in?

- Waikato
- Manawatu
- Canterbury
- West Coast

3. **How many animals** were you milking on the day of the screening visit?

| **Type** | **Number milked on date of screening** |
| --- | --- |
| Dairy heifers |  |
| Dairy cows (>2 years old) |  |

4. What type of milking shed do you have?

- rotary
- herringbone

**Lactation related**

5. What is the **major** calving season of your herd?

- spring
- autumn
- both

6. What is your planned start of calving?

_______________________

7. Can you estimate the average **herd milk production** (MS/cow year) approximately **in the last 12 months**?

________________________

**Cattle movement**

8. Do you have dairy cattle milking on **more than one farm**?

- yes, there is **movement** of these milking cattle between the farms I work on
- yes, but the cattle do not move
- no

9. Did you purchase any animals into your milking herd from outside **in the last 12 months**? If yes, where did you bring the following animals onto this farm?

| **Category** | **Major source of acquired animals** |
| --- | --- |
| Dairy heifers | - no I did not - directly from other farm(s) - sale yards |
| Dairy cows (>2 years old) | - no I did not - directly from other farm(s) - sale yards |
| Breeding bulls | - no I did not - directly from other farm(s) - sale yards |

10. Do your **calves** go away grazing off your farm(s)?

- yes mixed with calves from other farm(s)
- yes but not with calves from other farm(s)
- no

11. Do your **milking dairy cattle** go away grazing in winter?

- yes mixed with dairy cattle from other farm(s)
- yes but not with dairy cattle from other farm(s)
- no

12. Do you provide grazing for stock from other farms at your farm?

- yes
- no

13. Have you used a transport company to transport animals (not for slaughter) **in the last 12 months**?

- yes
- no

14. Do you share a loading ramp with another farm(s)?

- yes
- no

**Hoof care**

15. **Who** did most of the hoof trimming/ lame cattle treatment on your farm?

- yourself /farm staff
- vet
- hoof trimmer

16. Was hoof trimming equipment routinely **washed** with water between cattle?

- yes
- no

17. Was hoof trimming equipment routinely **chemically disinfected** between cattle?

- yes
- no

18. How often do you **use** a footbath?

- never
- sometimes
- the whole lactation

19. How often do you **change the contents** of the footbath?

______________________

**Farm management**

20. Which type of land did your lactating cattle access in winter or whenever the weather was poor (you can tick more than one option)?

- pasture
- stand-off pads, number of months / year_______________
- barns or cow houses, number of months / year_______________

21. What is the main material your cattle walk on to get from the paddock to the shed?

- pasture, mud
- stones
- concrete
- other ____________________

22. Did you use a feed pad?

- yes
- no

**Lameness history**

23. How many lame cows were on your farm **in the last 12 months**?

**_________________**

24. What was the most common reason for lameness on your farm **in the last 12 months**?

- white line disease
- foot rot
- sole damage
- other _________________
