# Supplementary material for: Bayesian analysis of herd-level risk factors for bovine digital dermatitis in New Zealand dairy herds
Source: BMC Vet Res. 2019 Apr 27;15:125. doi: 10.1186/s12917-019-1871-3 (PMC6487038; doi:10.1186/s12917-019-1871-3)
Supplement: Supplementary file 2 — OpenBUGS code for the three Bayesian multivariable models used in this study. (DOCX 19 kb) [file 12917_2019_1871_MOESM2_ESM.docx]

**Additional file 2 for Yang DA, Gates MC, Müller KR and Laven RA. ”Bayesian analysis of herd-level risk factors for bovine digital dermatitis in New Zealand dairy herds”. BMC Vet Research.**

***OpenBUGS code for multilevel mixed effects binomial model***

model{

for(k in 1:4) { u1[k] ~ dnorm(0, tau1) } #specify region level random effect

tau1<-1/sigma1/sigma1 #tau1 is the precision = 1/variance

sigma1~dunif(0,3) #sigma1 is the SD, prior for it

for(i in 1:127) {

y[i]~dbin(p[i],hs[i]) #lesion ~ binomial dist with prev and herd size

#heifer: buying heifers

#calfgrz: heifers co-grazing

#hf: lameness treated by outside staff

logit(p[i])<-b0+b[1]*heifer[i]+b[2]*calfgrz[i]+b[3]*hf[i]+u1[region[i]]+u2[i]

u2[i]~dnorm(0,tau2) #random effect at herd level

}

tau2<-1/sigma2/sigma2

sigma2~dunif(0,2) #same way as tau1, sigma1

#real priors

b0~dnorm(-7.600402, 0.712435) #logit(0.0005),0.0035

b[1]<-logitnorm[1]-b0

b[2]<-logitnorm[2]-b[1]-b0

b[3]~dnorm(0,0.001)

logitnorm[1]~dnorm(-6.21261, 1.03431) #0.002, 0.01

logitnorm[2]~dnorm(-5.2933, 0.819452) #0.005, 0.03

#diffuse priors

#for (j in 1:3){b[j]~dnorm(0,0.001)}

#b0~dnorm(-5,0.001)

#change1

#sigma1~dunif(0,5)

#sigma2~dunif(0,3)

#change2

#sigma1~dunif(0,9)

#sigma2~dunif(0,2)

#odds ratio

for (j in 1:3){or[j] <- exp( b[j] )}

}

y[] hs[] region[] calfgrz[] heifer[] hf[]

14 210 2 1 0 1

13 151 2 1 0 0

14 476 2 1 0 1

9 270 2 1 1 0

9 505 2 0 0 0

…

***OpenBUGS code for mixed effects beta regression model***

Model{

for (j in 1:3){w[j]~dnorm(0,tau)} #region level random effect

tau~dgamma(1,1) #place prior on precision

for (i in 1:100) {

z[i] ~ dbeta(alpha[i], beta[i]) #prob of DD lesion+ ~ beta dist

alpha[i] <- mu[i] * phi

beta[i] <- (1-mu[i]) * phi #re-parameterize alpha and beta using mu and phi

#regn: region, the rest same to the first model code

logit(mu[i]) <- b[1] + b[2]*calfgrz[i]+ b[3]*heifer[i]+w[regn[i]]

}

phi ~ dgamma(1,1) #prior for phi

for (i in 1:3){b[i]~dnorm(0,0.01)} #prior for regression coef

}

regn[] z[] calfgrz[] heifer[]

3 0.7421 1 0

3 0.2361 1 0

3 0.3018 0 0

3 0.9977 1 1

3 0.9987 1 1

...

***OpenBUGS code for fixed effect beta regression model***

Model{

phi ~ dgamma(1,1)

for (i in 1:5){b[i]~dnorm(0,0.01)}

for (i in 1:100) {

z[i] ~ dbeta(alpha[i], beta[i])

alpha[i] <- mu[i] * phi

beta[i] <- (1-mu[i]) * phi

#can and wai: dummy variables for Canterbury and Waikato regions

logit(mu[i]) <- b[1] + b[2]*calfgrz[i]+ b[3]*heifer[i]+b[4]*can[i]+b[5]*wai[i]

}

#pred prob

#manawatu

pr[1]<-ilogit(b[1])

pr[2]<-ilogit(b[1]+b[2])

pr[3]<-ilogit(b[1]+b[2]+b[3])

pr[4]<-ilogit(b[1]+b[3])

#can

pr[5]<-ilogit(b[1]+b[4])

pr[6]<-ilogit(b[1]+b[2]+b[4])

pr[7]<-ilogit(b[1]+b[2]+b[3]+b[4])

pr[8]<-ilogit(b[1]+b[3]+b[4])

#wai

pr[9]<-ilogit(b[1]+b[5])

pr[10]<-ilogit(b[1]+b[2]+b[5])

pr[11]<-ilogit(b[1]+b[2]+b[3]+b[5])

pr[12]<-ilogit(b[1]+b[3]+b[5])

}

z[] calfgrz[] heifer[] can[] wai[]

0.7421 1 0 0 1

0.2361 1 0 0 1

0.3018 0 0 0 1

0.9977 1 1 0 1

0.9987 1 1 0 1

…
