# Supplementary material for: Bayesian analysis of herd-level risk factors for bovine digital dermatitis in New Zealand dairy herds
Source: BMC Vet Res. 2019 Apr 27;15:125. doi: 10.1186/s12917-019-1871-3 (PMC6487038; doi:10.1186/s12917-019-1871-3)
Supplement: Supplementary file 3 — Results of the sensitivity analysis for Multivariable model 1. (DOCX 18 kb) [file 12917_2019_1871_MOESM3_ESM.docx]

**Additional file 3 for Yang DA, Gates MC, Müller KR and Laven RA. ”Bayesian analysis of herd-level risk factors for bovine digital dermatitis in New Zealand dairy herds”. BMC Vet Research.**

**Results of the sensitivity analysis for binomial model**

| **Model** | | | | | | |
| --- | --- | --- | --- | --- | --- | --- |
|  | mean | sd | MC_error | val2.5pc | median | val97.5pc |
| $\beta_{0}$ | -7.678 | 0.615 | 0.014 | -8.896 | -7.674 | -6.462 |
| $\beta_{1}$ | 1.327 | 0.400 | 0.006 | 0.548 | 1.324 | 2.126 |
| $\beta_{2}$ | 1.061 | 0.363 | 0.004 | 0.356 | 1.056 | 1.782 |
| $\beta_{3}$ | 0.782 | 0.417 | 0.005 | -0.038 | 0.780 | 1.605 |
| $\sigma_{U}$ | 2.303 | 0.460 | 0.002 | 1.331 | 2.359 | 2.968 |
| $\sigma_{W}$ | 1.425 | 0.172 | 0.001 | 1.119 | 1.414 | 1.795 |
| **Sensitivity analysis 1** | | | | | | |
|  | mean | sd | MC_error | val2.5pc | median | val97.5pc |
| $\beta_{0}$ | -8.076 | 1.236 | 0.064 | -10.650 | -8.054 | -5.707 |
| $\beta_{1}$ | 1.339 | 0.413 | 0.010 | 0.553 | 1.325 | 2.183 |
| $\beta_{2}$ | 1.097 | 0.386 | 0.006 | 0.355 | 1.091 | 1.867 |
| $\beta_{3}$ | 0.792 | 0.426 | 0.010 | -0.042 | 0.790 | 1.644 |
| $\sigma_{U}$ | 2.370 | 0.437 | 0.006 | 1.406 | 2.438 | 2.973 |
| $\sigma_{W}$ | 1.433 | 0.176 | 0.002 | 1.120 | 1.422 | 1.813 |
| **Sensitivity analysis 2** | | | | | | |
|  | mean | sd | MC_error | val2.5pc | median | val97.5pc |
| $\beta_{0}$ | -7.725 | 0.656 | 0.028 | -8.994 | -7.729 | -6.423 |
| $\beta_{1}$ | 1.331 | 0.405 | 0.010 | 0.528 | 1.334 | 2.123 |
| $\beta_{2}$ | 1.049 | 0.365 | 0.007 | 0.343 | 1.045 | 1.775 |
| $\beta_{3}$ | 0.756 | 0.422 | 0.010 | -0.064 | 0.756 | 1.596 |
| $\sigma_{U}$ | 3.222 | 0.953 | 0.005 | 1.510 | 3.200 | 4.884 |
| $\sigma_{W}$ | 1.431 | 0.179 | 0.002 | 1.119 | 1.417 | 1.820 |
| **Sensitivity analysis 3** | | | | | | |
|  | mean | sd | MC_error | val2.5pc | median | val97.5pc |
| $\beta_{0}$ | -7.572 | 0.651 | 0.027 | -8.840 | -7.571 | -6.294 |
| $\beta_{1}$ | 1.317 | 0.399 | 0.010 | 0.533 | 1.314 | 2.111 |
| $\beta_{2}$ | 1.085 | 0.368 | 0.007 | 0.375 | 1.082 | 1.827 |
| $\beta_{3}$ | 0.782 | 0.429 | 0.010 | -0.055 | 0.783 | 1.632 |
| $\sigma_{U}$ | 4.329 | 1.888 | 0.012 | 1.595 | 3.977 | 8.473 |
| $\sigma_{W}$ | 1.429 | 0.175 | 0.002 | 1.120 | 1.418 | 1.806 |
